# Non prescribed sale of antibiotics in Riyadh, Saudi Arabia: A Cross Sectional Study

**DOI:** 10.1186/1471-2458-11-538

**Published:** 2011-07-07

**Authors:** Aref A Bin Abdulhak, Mohamad A Altannir, Mohammed A Almansor, Mohammed S Almohaya, Atallah S Onazi, Mohammed A Marei, Oweida F Aldossary, Sadek A Obeidat, Mustafa A Obeidat, Muhammad S Riaz, Imad M Tleyjeh

**Affiliations:** 1 Internal Medicine Department, King Fahd Medical City, Aldabab Street, Riyadh, 11525, Saudi Arabia; 2 Research and Scientific Publication Center, King Fahd Medical City, Aldabab street, Riyadh, 11525, Saudi Arabia; 3 Alfaisal University, Takhassusi Street, Riyadh, 11533, Saudi Arabia; 4 Infectious Diseases Division, Mayo Clinic, 200 First Street S.W, Rochester, MN, 55905, USA; 5 Division of Epidemiology, Mayo Clinic, 200 First Street S.W, Rochester, MN, 55905, USA

## Abstract

**Background:**

Antibiotics sales without medical prescriptions are increasingly recognized as sources of antimicrobial misuse that can exacerbate the global burden of antibiotic resistance. We aimed to determine the percentage of pharmacies who sell antibiotics without medical prescriptions, examining the potential associated risks of such practice in Riyadh, Saudi Arabia, by simulation of different clinical scenarios.

**Methods:**

A cross sectional study of a quasi-random sample of pharmacies stratified by the five regions of Riyadh. Each pharmacy was visited once by two investigators who simulated having a relative with a specific clinical illness (sore throat, acute bronchitis, otitis media, acute sinusitis, diarrhea, and urinary tract infection (UTI) in childbearing aged women).

**Results:**

A total of 327 pharmacies were visited. Antibiotics were dispensed without a medical prescription in 244 (77.6%) of 327, of which 231 (95%) were dispensed without a patient request. Simulated cases of sore throat and diarrhea resulted in an antibiotic being dispensed in (90%) of encounters, followed by UTI (75%), acute bronchitis (73%), otitis media (51%) and acute sinusitis (40%). Metronidazole (89%) and ciprofloxacin (86%) were commonly given for diarrhea and UTI, respectively, whereas amoxicillin/clavulanate was dispensed (51%) for the other simulated cases. None of the pharmacists asked about antibiotic allergy history or provided information about drug interactions. Only 23% asked about pregnancy status when dispensing antibiotics for UTI-simulated cases.

**Conclusions:**

We observed that an antibiotic could be obtained in Riyadh without a medical prescription or an evidence-based indication with associated potential clinical risks. Strict enforcement and adherence to existing regulations are warranted.

## Background

Antibiotic sales without medical prescriptions have been observed in many countries [1-5]. This exacerbates the existing problem of inappropriate use of antibiotics that leads to an increase in treatment cost, drug adverse effects, and antibiotic resistance among bacteria [6].

Antibiotic resistance is a global health problem, closely related to volume of antibiotic consumption [7,8]; therefore, restricting antibiotic use and marketing regulations are among many important strategies to control this problem [9,10].

However, most initiatives regarding antibiotic misuse are directed toward optimizing physicians' prescriptions [11-16], while other potential sources of antibiotic misuse are neglected.

It has been illegal for pharmacists in Saudi Arabia to dispense an antibiotic without a medical prescription for more than three decades [17]. However, a previous study from the Eastern Province of Saudi Arabia demonstrated a high rate of antibiotic sales without a prescription for presumed urinary tract infections [18] due to lack of adherence to these regulations.

Therefore, we sought to determine the percentage of pharmacies who sell antibiotics without a medical prescription, exploring the potential associated risks of this practice in Riyadh, Saudi Arabia, by simulation of six different clinical scenarios.

## Methods

A cross sectional study of a quasi-random sample of 327 pharmacies was conducted in Riyadh, the capital of Saudi Arabia with about 5 million habitants, in November 2010. The sample was intended to be representative of all Riyadh pharmacies. The sample was stratified by the five regions of Riyadh (Eastern, Western, Northern, Southern, Central) regardless of the pharmacy's size, deprivation level of the area. A convenience sample of streets was chosen from each region and a complete enumeration of all pharmacies in each street was considered. Each pharmacy was visited once by two investigators (total of 6 male physicians and 2 male medical students participated) who simulated having a brother/sister with a predetermined clinical scenario according to simulated-client method pharmacy surveys [19,20]. The scenarios included sore throat, acute bronchitis, otitis media, acute sinusitis, diarrhea, and urinary tract infection in a pregnant (childbearing age) women. The investigators concealed their identity and the study objective of their visits from the approached pharmacists who were identified by their licenses and pictures on the front wall of the pharmacy. The clinical scenarios were presented as follow; one investigator talked to the pharmacist while the other observed the discussion and memorized the responses. Immediately after leaving the pharmacy, both investigators completed a standardized data form that included information about the location of the pharmacy, antibiotics dispensing practice, pharmacists' inquiries about associated symptoms (e.g. fever/shortness of breath/abdominal pain/loin pain), allergy history, pregnancy status in case of UTI; type of antibiotic, if dispensed; and information about drug interactions if this was provided by the pharmacist.

Two sessions of standardization took place in the presence of all actors. Each group rehearsed simulating all the clinical scenarios to the senior investigator using the same complaints (terminology and statements). Rehearsal was repeated to ensure reliability of the simulated scenario. The actors used lay language and refrained from using any jargon.

Only the following clinical information was presented to the pharmacist. Any additional information was only provided if the pharmacist inquired about it. The sore throat scenario: a healthy young male relative was described as having difficulties in swallowing with slight fever of 24 hours duration. Acute bronchitis scenario: an elderly man relative was described as having sore throat, cough with sputum production. Additional information provided upon request was the patient had multiple comorbid conditions and was using warfarin.

Acute sinusitis scenario: a young male relative was described as having running nose, facial pain, and headache. Otitis media scenario: a 5-year-old relative child was described as having ear pain and discharge. Urinary tract infection scenario: a childbearing female relative was described as having dysuria and urinary frequency. Diarrhea scenario: a young male relative was described with loose bowel motion for one day.

Three levels of demand were used sequentially until an antibiotic was dispensed or denied [4]: 1) Can I have something to relieve my symptoms?: 2) Can I have something stronger? 3) I would like to have an antibiotic.

Data are presented as percentage of the pharmacists' responses toward the simulated clinical scenarios.

The study was approved by the Institutional Review Board at King Fahd Medical City. Deception and incomplete disclosure to study subjects (pharmacists) were considered ethically acceptable because this was a minimal risk study and it could not have been performed with complete disclosure of investigator entity. Data were kept anonymous.

## Results

Three hundred twenty-seven pharmacies were visited from all five regions of Riyadh (Eastern, Western, Northern, Southern, Central).

Antibiotics were dispensed without medical prescription from 244 (77.6%) of 327 with different levels of demand. Simulated cases of sore throat and diarrhea accounted for the highest percentage of antibiotic sales without medical prescription (90%) with level one of demand, followed by UTI (75%), acute bronchitis (73%), otitis media (51%) and acute sinusitis (40%).

The distribution of the percentage of pharmacies that dispensed antibiotics without prescription with different levels of demand for the simulated scenarios is summarized in Table 1.

**Table 1:** Percentage of Pharmacies Willing to Sell Antibiotics without Medical Prescription According to Strength of Patient Demand

Level of demand	Sore throat n = 58	Acute sinusitis n = 55	Otitis media n = 53	Acute bronchitis n = 44	Diarrhea n = 59	UTI n = 58	All n = 327
**Level one**	53 (90%)	22(40%)	27(51%)	32(73%)	53(90%)	44(75%)	231(70%)
**Level two** **or three**	0(0%)	16(29%)	0(0%)	3(7%)	4(7%)	0(0%)	23(7%)
**All level s**	53(90%)	38(69%)	27(51%)	35(80%)	57(97%)	44(75%)	254(77.6%)
**Evidence based ** ** appropriateness ** ** of antibiotics use ** ** [Reference]**	Antibiotics use confer relative benefits [28]	No[29]	Antibiotics use confer small benefits[30]	No [31]	No[32]	Yes[33]	

None of the visited pharmacists asked about history of drug allergy or provided information regarding potential drug interactions when dispensing any antibiotics for any of the simulated clinical scenarios.

Amoxicillin/clavulanate was the most commonly dispensed antibiotic in cases of sore throat, acute bronchitis, acute sinusitis, and otitis media whereas ciprofloxacin and metronidazole were commonly given for UTI and diarrhea cases, respectively.

Only 23% of pharmacists who dispensed antibiotics for UTI simulated cases inquired about pregnancy status.

The majority of pharmacists did not inquire about associated symptoms, and only few recommended a medical evaluation. Table 2 summarizes the main interview aspects and the dispensed antibiotics across different case scenarios.

**Table 2:** Pharmacists' Inquiries and Recommendations in Response to the Simulated Clinical Scenarios

Simulated clinical scenario	Recommended medical evaluation (%) (95% CI)	Asked about associated symptoms (%) (95% CI)	Asked about presence of fever (%) (95% CI)	Asked about pregnancy status (%) (95% CI)	Dispensed antibiotic
**Sore throat** **(n = 58)**	5% (1.1%-14.4%)	40% (27.0% -53.4%)	9% (2.9%-19.0%)	NA	Amoxicillin/Clavulanate (60%) Azithromycin (20%) Amoxicillin (17%) Others (3%)
**Acute bronchitis** **(n = 44)**	14% (5.2%-27.4%)	43% (28.3%-59.0%)	25% (13.2%-40.3%)	NA	Amoxicillin/Clavulanate (42%) Amoxicillin (27%) Azithromycin (15%) Others (16%)
**UTI** **(n = 58)**	10% (3.9%-21.2%)	22% (12.5%-35.3%)	14% (6.1%-25.4%)	23% (12.5%-35.3%)	Ciprofloxacin (86%) Amoxicillin (7%) Cefixime (2%) Clarithromycin (2%) Others (3%)
**Diarrhea** **(n = 59)**	3% (0.4%-11.7%)	62% (49.1%-75.0%)	14% (6.0%-23.0%)	NA	Metronidazole (89%) Ciprofloxacin (5%) Cotrimoxazole (2%) Ofloxacin (2%) Others (2%)
**Acute sinusitis** **(n = 55)**	2% (0.0-9.7%)	73% (59.0%-83.9)	9% (3.0%-20.0%)	NA	Amoxicillin/Clavulanate (58%) Azithromycin (26%) Amoxicillin (8%) Cefaclor (3%) Ofloxacillin (3%) Others (2%)
**Otitis media** **(n = 53)**	47% (33.3% -61.4%)	19% (8.3%-29.4%)	2% (0.0-10.1)	NA	Amoxicillin/Clavulanate (44%) Amoxicillin (29%) Cephalexin (15%) Azithromycin (4%) Cefaclor (4%) Cefixime (4%)

NA: Not applicable CI: Confidence interval

## Discussion

In this representative sample of community pharmacies in Riyadh, we observed that antibiotic could be easily obtained without a medical prescription or an evidence-based indication. These scenarios represent prevalent diseases that are commonly due to viral infections with the exception of UTI and otitis media. Despite this fact, most pharmacists were more likely to provide antibiotics inappropriately since 97% of diarrhea received antibiotics versus 75% of UTI. Moreover, pharmacists dispensed broad spectrum antibiotics without even being requested by the actors (level one demand).

Amoxicillin/clavulanate was the most commonly prescribed antibiotic for presumed cases of sore throat, acute sinusitis, acute bronchitis, and otitis media. Additionally, ciprofloxacin was given frequently for presumed UTI in a childbearing age women without verifying the pregnancy status before dispensing this antibiotic which is FDA class C agent in pregnancy.

Moreover, none of the interviewed pharmacists asked about allergy history or provided information about potential drug interactions which may lead to a greater risk.

The high observed rate of antibiotic sales without a prescription in Riyadh could be explained by several factors: lack of enforcement of the national regulations, suboptimal compliance to the code of ethics and professionalism among community pharmacists, and financial interests of community pharmacists [17].

In a systematic review by Morgan et al [21] of 35 community surveys from five continents, non-prescription use of antibiotics occurred worldwide and accounted for 19-100% of antimicrobial use outside of northern Europe and North America. Similar to our findings, Morgan et al observed that non-prescription use was common for non-bacterial disease. In 2 of the 35 surveys, more than 80% of pharmacists did not inquire about drug allergy [21]. There was a wide variation between studies results which could be attributable to variations in the clinical scenarios [21]. Other factors which may be responsible for the variations between studies are level of pharmacists training, and variations in adherence to the existing regulations.

Obtaining antibiotics without prescription will not only promote antimicrobials resistance but can also be associated with significant adverse events including drug adverse effects, high cost and complications [6].

Antimicrobial resistance is a major concern associated with this practice. Memish et al [22] has documented an increase in resistance to penicillin among all strains of *Streptococcus pneumoniae *in Saudi Arabia. Our findings have shown, for example, inappropriate use of amoxicillin/clavulanate that can contribute to the selective pressure and selection of resistance to this class of antibiotic that is usually held "in reserve".

Adverse drug reactions represent another major concern. In a meta-analysis of 39 prospective studies from US hospitals, the overall incidence of serious adverse drug reactions (ADRs) was 6.7% (95% confidence interval [CI], 5.2%-8.2%) and of fatal ADRs was 0.32% (95% CI, 0.23%-0.41%) of hospitalized patients [23].

In another analysis of drug-related adverse events from the National Electronic Injury Surveillance System-Cooperative Adverse Drug Event Surveillance project (2004-2006) and outpatient prescriptions from national sample surveys of ambulatory care practices, the National Ambulatory Medical Care Survey and the National Hospital Ambulatory Medical Care Survey (2004-2005), an estimated 142,505 visits (95% confidence interval [CI], 116,506-168,504 visits) annually were made to US Emergency Departments (EDs) for drug-related adverse events attributable to systemic antibiotics. Antibiotics were implicated in 19.3% of all ED visits for drug-related adverse events. Most ED visits for antibiotic-associated adverse events were for allergic reactions (78.7% of visits; 95% CI, 75.3%-82.1% of visits) [24].

In addition, serious drug interaction is a major sequalea. Amoxicillin/clavulanate, for example, interacts with warfarin and lead to an increasing bleeding risk. Failure of pharmacists to ask patients about concomitant medications poses a significant risk to patients.

Moreover, pregnancy status is a critical situation to consider when giving any medication to a child bearing woman. In our simulated case scenario, a pregnant woman could have been exposed to a Class C medication(Ciprofloxacin); while the evidence supports avoiding this drug during pregnancy because of the difficulty in extrapolating animal mutagenicity results to humans and interpretation of the toxicity is still controversial[25].

Parasitic infestation is not highly prevalent in Riyadh [26]. However, metronidazole was dispensed, based on no supporting evidence to most simulated cases of diarrhea. Metronidazole can cause serious side effects such as seizure, encephalopathy, and peripheral neuropathy [27].

Future studies should examine reasons why patients seek non-prescribed medications directly from pharmacists despite the presence of public hospitals and primary care centers.

Our study findings may not be generalizable to other countries. Although the actors were medical professionals, they have not used medical jargon to avoid introducing bias in favor of antibiotic sale without prescription.

## Conclusion

In a representative sample of community pharmacies in Riyadh, we observed that antibiotics could be easily obtained without a medical prescription or an evidence-based indication. There are major potential sequalae associated with this practice. There is a need for strict enforcement and adherence to existing regulations regarding antibiotics sale. Educating the public about the worldwide existing problems of antibiotic resistance, drug adverse effects and unnecessary cost associated with antibiotic sales without medical prescription is urgently needed.

## Competing interests

The authors declare that they have no competing interests.

## Authors' contributions

ABA contributed in acquisition, analyzing and interpretation of data; drafting and revising the manuscript and approved the final version of manuscript for publication. MAA contributed to analyzing and interpretation of data; drafting and revising the manuscript and approved the final version of manuscript for publication. MAM, MSM, ANO, OFD, SAO, MAO, MSR contributed in acquisition, analyzing and interpretation of data; approved the final version of manuscript for publication. 

IMT made the concept and designed study, analyzed and interpreted the data, revised and approved the final version of manuscript for publication

## Pre-publication history

The pre-publication history for this paper can be accessed here:

http://www.biomedcentral.com/1471-2458/11/538/prepub
